# SARS-CoV-2 Spike Protein and Molecular Mimicry: An Immunoinformatic Screen for Cross-Reactive Autoantigen Candidates

**DOI:** 10.3390/ijms26188793

**Published:** 2025-09-10

**Authors:** Anna M. Timofeeva, Kseniya S. Aulova, Egor A. Mustaev, Georgy A. Nevinsky

**Affiliations:** 1 SB RAS Institute of Chemical Biology and Fundamental Medicine, Novosibirsk 630090, Russia; 2 SRF “SKIF”, Koltsovo 630559, Russia; 3 Department of Natural Sciences, Novosibirsk State University, Novosibirsk 630090, Russia

**Keywords:** molecular mimicry, autoreactivity, epitopes, autoimmunity, autoimmune disease, viral infection, SARS-CoV-2, COVID-19, autoantibodies, HLA, MHC, B cell, T cell, immunoinformatics

## Abstract

This study investigated the role of molecular mimicry in the context of autoimmunity associated with viral infection, using SARS-CoV-2 as a model system. A bioinformatic analysis was performed to identify sequence homologies between the SARS-CoV-2 Spike (S) protein and the human proteome, with a specific focus on immunogenic regions to assess potential cross-reactivity. The analysis revealed homologous regions between the viral S protein and several human proteins, including DAAM2, CHL1, HAVR2/TIM3, FSTL1, FHOD3, MYO18A, EMILIN3, LAMP1, and αENaC, which are predicted to be recognizable by B-cell receptors. Such recognition could potentially lead to the production of autoreactive antibodies, which can contribute to the development of autoimmune diseases. Furthermore, the study examined potential autoreactive CD4+ T-cell responses to human protein autoepitopes that could be presented by HLA class II molecules. Several HLA class II genetic variants were computationally associated with a higher likelihood of cross-reactive immune reactions following COVID-19, including HLA-DPA1*01:03/DPB1*02:01, HLA-DPA1*02:01/DPB1*01:01, HLA-DPA1*02:01/DPB1*05:01, HLA-DPA1*02:01/DPB1*14:01, HLA-DQA1*01:02/DQB1*06:02, HLA-DRB1*04:01, HLA-DRB1*04:05, HLA-DRB1*07:01, and HLA-DRB1*15:01. Additionally, seven T helper cell autoepitopes (YSEILDKYFKNFDNG, ERTRFQTLLNELDRS, AERTRFQTLLNELDR, RERKVEAEVQAIQEQ, NAINIGLTVLPPPRT, PQSAVYSTGSNGILL, TIRIGIYIGAGICAG) were identified that could be implicated in autoimmune T-cell responses through presentation by class II HLA molecules. These findings highlight the utility of viral B- and T-cell epitope prediction for investigating molecular mimicry as a possible mechanism in virus-associated autoimmunity.

## 1. Introduction

Viral infections have been associated with the onset or exacerbation of autoimmune diseases through several proposed mechanisms [1]. One key pathway is molecular mimicry, which occurs when there is a structural similarity between viral and host peptides. This phenomenon can activate naïve autoreactive T or B cells [2]. This cross-reactivity can lead to a shift in the immune response, causing the immune system to attack itself, resulting in the development of autoimmune diseases [3].

A critical aspect of immunological viral protein recognition is the capacity of the human immune system to differentiate foreign proteins (such as SARS-CoV-2 proteins) from its own proteins [4,5]. Theoretically, the immune system should not produce antibodies against its own proteins. However, certain viruses have evolved to have human-like sequences, which help them evade detection by the immune system [6,7,8]. As a result, numerous viral infections have been linked to the formation of autoreactive antibodies through molecular mimicry between human and viral proteins. For example, infection with the Epstein–Barr virus has been linked to an over-30-fold increased risk of multiple sclerosis [9,10,11,12]. Also, antibodies targeting the Epstein–Barr virus nuclear antigen were found to react with glial cell adhesion protein in the central nervous system (CNS) [13]. Additionally, a shared sequence was found between Coxsackie virus nonstructural protein 2C and glutamic acid decarboxylase 65 (GAD65), a protein preferentially expressed in pancreatic beta cells [14]. GAD65 is known to be a significant factor in the development of type 1 diabetes [15]. Patients with type 1 diabetes have been found to develop antibodies that cross-react with both GAD65 and protein 2C [16].

A recent study analyzing proteins from five viruses (Borne disease virus, HIV, measles, mumps, and rubella) compared them with the human proteome and revealed extensive sequence overlap, which could lead to the creation of autoreactive antibodies through molecular mimicry [17]. Furthermore, numerous reports detail instances of autoimmune disorders like multisystem inflammatory syndrome, Guillain–Barré syndrome, lupus, type 1 diabetes, and rheumatoid arthritis occurring after COVID-19 infection [18,19,20,21], which may be related to molecular mimicry [22].

Some studies have identified similarities between SARS-CoV-2 protein sequences and human proteins [23,24,25,26]. The development of autoreactive antibodies is an undesirable outcome of infections or vaccinations, making the analysis of the autoimmune potential of viral proteins a significant area of scientific inquiry. Multi-epitope vaccine development necessitates careful examination of viral epitopes to exclude any that show homology with human proteins.

This study employed bioinformatic approaches to investigate potential molecular mimicry between the SARS-CoV-2 S protein and human proteins by analyzing short compound sequences (SCSs) of amino acids. The S protein sequence was divided into heptapeptides displaced relative to each other by one amino acid residue. The resulting heptapeptide library was screened for sequence homology against the human proteome. It should be noted that identified sequence homologies do not necessarily indicate biological relevance, as structural constraints or subcellular localization may prevent these regions from functioning as actual epitopes. To identify SCS sequences capable of eliciting an immune response, B-cell epitope prediction was conducted for each selected human protein exhibiting sequence homology. Our computational analysis identified surface-accessible regions in nine human proteins that exhibit sequence similarity to the SARS-CoV-2 S protein and are predicted to be potentially recognizable by B-cell receptors based on bioinformatic criteria.

Additionally, we performed in silico analysis of CD4+ T-cell reactivity to predicted human helper T lymphocyte (HTL) protein epitopes through computational prediction of HLA class II binding affinity. Our bioinformatic assessment identified nine specific HLA class II alleles that are predicted to potentially bind autoepitopes and seven HTL epitopes that may be presented through HLA class II molecules. All results presented herein are derived from predictive bioinformatic models and should be interpreted as preliminary computational insights only.

## 2. Results

### 2.1. Predicting B Antigen Recognition by B Cells

**Figure 1:**
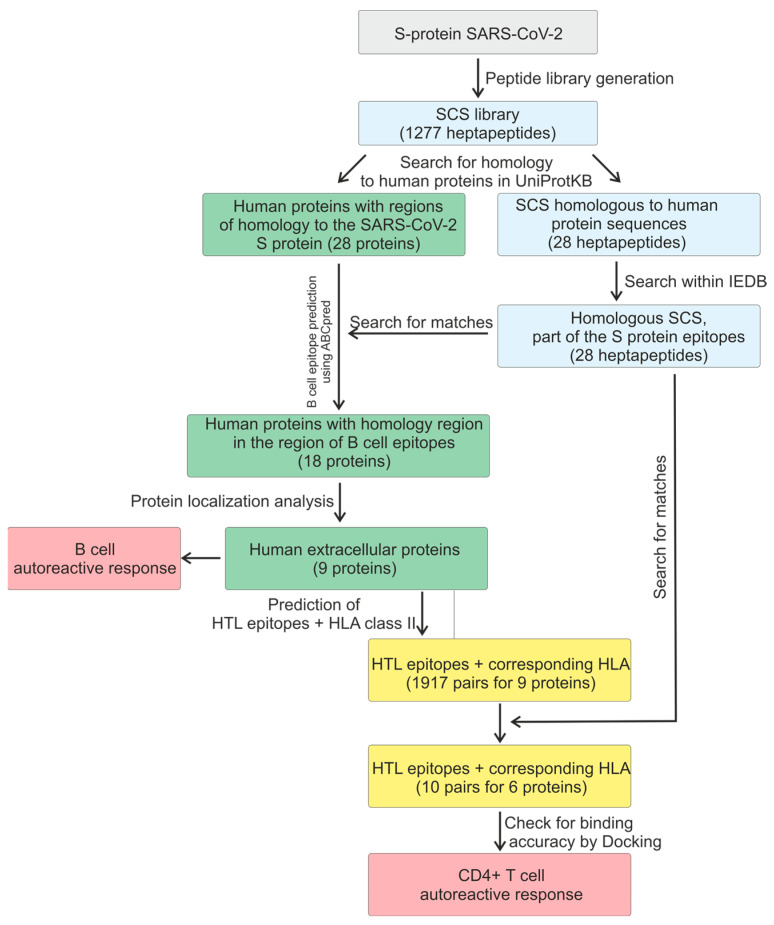
The stages of the research.

The methodology of this study encompasses several stages of data acquisition to assess the autoimmune response-triggering potential of the SARS-CoV-2 proteome. A summary of these stages is presented in Figure 1.

The S protein amino acid sequence (Wuhan strain) was partitioned into overlapping heptapeptides (SCS). A total of 1277 SCSs were compiled and underwent screening for their inclusion in the human proteome. This process identified 28 proteins with sequence similarity to the S protein (Table S1).

Five viral variants underwent multiple amino acid sequence alignment analysis to identify conserved Spike regions. The conservativity of 28 heptapeptides was tested. All identified oligopeptides, excluding NSPRRAR, resided in conserved domains of the S protein.

It is noteworthy that SCSs may be situated within or outside immunogenic epitopes. Cross-reactivity is primarily determined by peptides residing within immunogenic epitopes [27,28,29]. The number of potential triggers of autoimmune processes may be reduced by considering the location of the peptide within the protein structure. Given the above, our investigation comprised (1) an examination of the location of the selected SCSs in the viral S protein structure and (2) an assessment of the availability of homologous human protein sequences for immune system recognition. To clarify, for every heptapeptide, we investigated its simultaneous presence within the S protein and B-cell epitopes of the human protein.

To answer the question of whether heptapeptides are part of viral protein epitopes, the sequence matches of S protein heptapeptide sequences were analyzed with sequences downloaded from the experimentally validated Immune Epitope Database (IEDB) [30]. All 28 heptapeptides were represented in the IEDB database, indicating that all 28 heptapeptides can be recognized by the immune system upon infection with the SARS-CoV-2 virus.

The next step involved employing the ABCpred resource to predict B-cell epitopes within twenty-eight human proteins. The frequency of heptapeptides within the predicted epitope set was assessed. Table 1 presents human proteins containing heptapeptide sequences located within epitope regions, potentially eliciting B-cell recognition and autoreactive immune responses. Eighteen human proteins with identifiable regions of homology to the S protein have been found to be recognized by B cells within the immune system.

Subsequent consideration should be given to the likelihood of autoantigens interacting with autoreactive antibodies and B-lymphocytes. Intracellular proteins, including those within the nucleus, cytoplasm, or membranes, typically evade immune system recognition. Extracellular proteins can be the target of autoreactive antibodies. Therefore, UniProtKB data and literature data were further analyzed to establish the extracellular or intracellular localization of the identified 18 proteins. The membrane topology of predicted epitopes on membrane-bound proteins was investigated. For example, the luminally located epitopes of interest in the membrane proteins LAMP1 and αENaC (encoded by the *SCNN1A* gene) were documented (https://www.uniprot.org/uniprotkb/P11279/entry, https://www.uniprot.org/uniprotkb/C5HTY8/entry accessed on 17 January 2025). And the ABCA10 protein epitope of interest is located inside the membrane. Thus, of the 18 proteins, only 9 proteins can interact with the immune system: DAAM2, CHL1, HAVR2/TIM3, FSTL1, FHOD3, MYO18A, EMILIN3, LAMP1, and αENaC (Table 1, column “Protein localization”).

HAVR2, also known as T-cell immunoglobulin containing mucin domain-3 (TIM-3), is an immune checkpoint receptor on the surface of T cells [31,32,33]. Additionally, this receptor is located on the surface of innate immune system cells [34]. HAVR2/TIM-3 contributes to immune equilibrium and minimizes tissue damage stemming from an overactive inflammatory response [35,36]. Additionally, it modulates macrophage activation [33], suppresses auto- and alloimmune responses, and promotes immunologic tolerance [37]. Notably, the therapeutic potential of immune checkpoint inhibitors in cancer treatment is of significant importance. Antibodies targeting immune checkpoints, including TIM-3, are currently in development [38]. However, the use of immune checkpoint receptor inhibitors in cancer therapy is limited due to the development of autoimmune-like immune-related adverse events [39]. Preclinical research demonstrates that TIM-3 antagonism is associated with augmented disease severity in the context of autoimmune inflammation [33,37].

*Myo18A* gene expression yields diverse protein isoforms through alternative splicing, exhibiting cell-type specificity [40]. Myo18Aα plays a crucial role in regulating innate immune functions of macrophages and B-cell homeostasis [41,42] and participates in the activation of the inflammatory response, including the production of inflammatory cytokines [43,44,45].

Secreted glycoprotein FSTL1 has been shown to participate in several key physiological processes: angiogenesis, modulation of the immune response, and cell proliferation and differentiation [46,47]. Studies using murine models revealed a positive correlation between increased FSTL1 expression and improved graft engraftment [48]. Consequently, the generation of antibodies targeting TIM3, Myo18A, and FSTL1 may compromise immune tolerance, potentially resulting in autoimmune responses.

Human epithelial sodium channels (ENaCs) are heterotrimeric structures, each comprising alpha, beta, and gamma subunits. The *SCNN1A* gene encodes the α ENaC subunit (αENaC) [49]. ENaC is responsible for limiting the rate of Na^+^ reabsorption and plays an important role in maintaining sodium homeostasis, extracellular fluid volume, and blood pressure [50]. Within endothelial cells, ENaC plays a crucial role in maintaining the alveolar-capillary barrier. The pathogenesis of SARS-CoV, SARS-CoV-2, and MERS-CoV infections involves respiratory distress stemming from fluid-induced impairment of gas exchange and subsequent alveolar edema [51,52,53]. A potential etiology of disrupted water homeostasis is the compromised ion transport capabilities of the lung epithelium, specifically the reduction in sodium reabsorption due to ENaC inhibition [54,55]. The expression of epithelial sodium channels (ENaC) within taste receptor cells of the tongue is critical for salt taste transduction. The presence of Na^+^ induces ENaC-mediated depolarization of taste receptor cells, leading to salt sensation [56]. Therefore, reduced ENaC activity is linked to a decrease in taste perception. It is noteworthy that the loss of smell and taste are among the most prevalent symptoms reported in SARS-CoV-2 cases [57,58]. The alpha subunit of the epithelial sodium channel (αENaC) has previously been shown by in silico methods to contain a motif that is 100% identical to the furin cleavage site of the S protein of SARS-CoV-2 virus [59]. Consequently, the possibility that SARS-CoV-2–infected patients generate anti-αENaC antibodies contributing to COVID-19 symptom presentation cannot be excluded.

Post-COVID-19 patients frequently exhibit neurological and neuropsychiatric manifestations for extended periods following infection [60,61]. Access of circulating immune cells to the CNS is limited in healthy individuals under steady-state conditions, with only very few lymphocytes and non-resident myeloid cells in the brain parenchyma. However, in inflammatory diseases, the number of immune cells entering the CNS parenchyma through the blood–brain barrier or the barrier between blood and cerebrospinal fluid increases dramatically [62,63,64,65,66]. A wide range of phenotypes and effector functions are displayed by T and B cells during infiltration, impacting both disease protection and pathogenesis [67,68]. B cells are involved in the pathogenesis of neuroinflammatory diseases, including autoimmune diseases, through various mechanisms, such as antigen presentation to T cells [69], antigen transport to secondary lymphoid organs, secretion of pro-inflammatory or anti-inflammatory cytokines [70,71], and pathogenic antibodies [68,72]. Autoimmune antibodies to various protein targets, such as myelin oligodendrocyte glycoprotein (MOG) [73,74,75], major basic protein (MBP) [76,77,78], dipeptidyl-peptidase-like protein 6 (DPPX) [79,80], and aquaporin-4 [81,82], have been described and are even applied in the diagnosis of certain neurological disorders [83,84]. The occurrence of neurological impairment in individuals following SARS-CoV-2 infection suggests a potential contribution from the immune response. Such disorders have been shown to correlate with the presence of autoantibodies to contactin-associated protein 2 (Caspr2), ganglioside GD1b, myelin oligodendrocyte glycoprotein, and the major protein of myelin [85,86,87,88]. Therefore, we specified two proteins associated with the nervous system and having extracellular localization: DAAM2 and CHL1.

DAAM2, a formin family protein, participates in the assembly of both actin filaments and microtubules [89]. DAAM2 is critical for regulating oligodendrocyte morphology, myelin structure, and remyelination [90,91]. The neural cell adhesion protein CHL1 contributes to the development of the nervous system and synaptic plasticity in the mature nervous system. Both soluble and membrane-associated variants enhance neurite extension in cerebellar and hippocampal neurons and concurrently inhibit neuronal death [92]. Chl1-deficient mice were demonstrated to exhibit impaired synaptic transmission, long-term potentiation, working memory performance, sensorimotor processing, and prepulse inhibition of acoustic startle response [93,94,95,96]. It is not inconceivable that, subsequent to SARS-CoV-2 infection, autoantibodies against the DAAM2 or CHL1 proteins can be produced that may contribute to the neurological manifestations of COVID-19.

The expression of FHOD3 is primarily observed in transversely striated muscles and cardiomyocytes [97,98,99,100]. FHOD3 is also a member of the formin family and is responsible for the assembly of actin filaments in cardiomyocyte sarcomeres.

EMILIN3 is a glycoprotein of the extracellular matrix. There is evidence that this protein can also affect TGF-β1 cytokine signaling by binding to its precursor protein (pro-TGF-β1) [101]. Chronic COVID-19 patients exhibited a marked decrease in exercise tolerance, which was significantly associated with metabolic changes in skeletal muscle. Post-load malaise pathophysiology involves the reduction in acute exercise-induced skeletal muscle mitochondrial enzyme activity, increased skeletal muscle amyloid deposition, evident muscle tissue damage, and a blunted skeletal muscle T-cell response [102]. It is plausible that autoreactive responses potentially linked to viral protein mimicry are involved in these processes.

LAMP-1 constitutes a critical structural element within lysosomes [103]. It plays a regulatory role in lysosome exocytosis [104,105], promotes protein degradation via the lysosomal pathway, and transports antigen to the MHC class II compartment, resulting in activation of CD4+ helper T cells [106,107,108,109]. Additionally, LAMP1 is implicated in the viral life cycle of endosomally entering viruses, including SARS-CoV-2 [110], Lassa virus [111], and hantaviruses [108,112]. LAMP1 allows viral particles to exit the endocytic pathway before they encounter a more acidic proteolytic environment, resulting in more viable virions. Consequently, the interaction of LAMP-1 and the virus plays a significant role in viral infectivity. The action of autoreactive antibodies on this protein could limit the propagation of viral particles and enhance healing processes.

Therefore, our computational analysis identified surface-accessible regions in nine human proteins that exhibit sequence similarity to the SARS-CoV-2 S protein and are potentially recognized by B-cell receptors. These proteins could theoretically act as autoantigens, potentially leading to the development of autoimmune conditions.

### 2.2. T-Cell-Dependent Antibody Response

Autoimmune diseases are characterized by the loss of self-tolerance with the activation of autoreactive B cells as well as T cells [113,114]. The mechanism of autoreactive T-cell response in the case of molecular mimicry is also considered here.

The activation of CD4+ T cells, or helper T lymphocytes, is significantly influenced by B-cell-mediated antigen presentation [115,116,117]. B cells exhibiting antigen specificity utilize their B-cell receptors to bind, internalize, and subsequently process antigens into smaller peptide fragments. These epitopes are expressed on the plasma membrane in complex with MHC class II molecules for recognition by CD4+ T cells via the T-cell receptor [118,119]. CD4+ T cells subsequently promote B-cell proliferation [120].

Thus, when analyzing the possibility of an autoreactive T-cell response during molecular mimicry, account should be taken of the possibility of peptide binding not only to the T-cell receptor but also to MHC class II molecules, which in humans are also called human leukocyte antigen (HLA) class II [121].

SARS-CoV-2 S protein binds to B-cell receptors, undergoes internalization and processing, yielding peptides recognizable by T cells. We examined the influence of HTL epitopes on the development of autoreactive responses. For this purpose, we analyzed the homology of HTL epitopes of the S protein with human proteins and the potential for peptide loading onto HLA class II molecules. The HTL epitopes of nine human proteins were predicted using the MHC class II binding module available on the IEDB server. This module is designed to detect 15-mer epitopes and their corresponding HLAs. A consensus percentile rank score ≤ 2.0 served as the predictive threshold for effective HTL epitopes [122,123]. The predicted HTL epitopes underwent testing for the incorporation of homologous heptapeptides. The results are summarized in Table 2. Only six proteins were found to bind the heptapetide S protein homologous sequence to the corresponding MHC II: DAAM2, HAVCR2, FHOD3, CHL1, EMILIN3, and FSTL1. Notably, the DAAM2 protein sequence is predicted to be recognized by five distinct HLA class II alleles, while the remaining proteins exhibit predicted recognition by only a single HLA allele. The analysis was conducted using a representative panel of the most prevalent HLA class II genes [124,125]. This study omits an analysis of other allelic variants that may be present within the population. However, given that closely related HLA allelic variants were previously shown to be characterized by an overlapping binding repertoire [126,127,128], our analysis can be considered comprehensive.

The HLA system demonstrates a high degree of polymorphism, with thousands of allelic variants documented in the human population [121]. Polymorphic residues are predominantly located in the peptide-binding groove, thereby generating unique binding patterns for each allelic variant. Variations in HLA genes correlate with observed patterns of peptide recognition within populations. Peptide population coverage may be restricted by the infrequent occurrence or absence of specific HLA alleles. Research on MHC class II genetic variants has not been conducted comprehensively across diverse populations. Consequently, all identified HLA alleles underwent further analysis, irrespective of variant rarity. It should be noted that HLA-DRB1*04:01, HLA-DRB1*07:01, and HLA-DRB1*15:01 alleles are among the Top 10 HLA-DRB1 allele frequencies by geographic region in Europe, and HLA-DRB1*04:05 alleles are in Northeast Asia (data from http://www.allelefrequencies.net/, accessed 23 January 2025). Subsequently, molecular docking of fifteen-residue peptides, specifically HTL epitopes, with their cognate HLA-II molecules, was conducted.

### 2.3. Molecular Docking of HTL Epitopes with HLA-II

**Figure 2:**
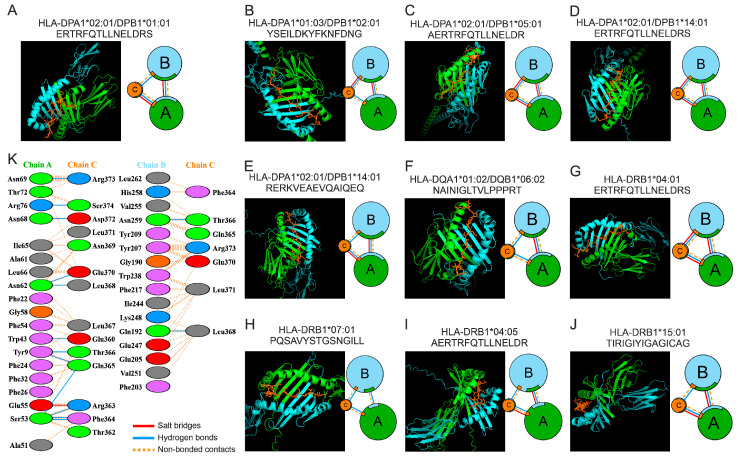
(**A**–**J**) The binding interface between HTL epitopes and HLA class II molecules. Visualization of the molecular docking results was achieved with PyMOL 3.1.3. The protein–protein interface between (**A**,**B**) (HLA) chains and the (**C**) (HTL epitope) chain exhibits interacting amino acid residues, as shown by PDBsum data. The area of each circle is directly proportional to the surface area of the associated protein chain. The extent of the interface region on each chain is represented by the black wedge, whose size signifies the interface surface area. (**K**) The amino acids involved in intermolecular contact formation of HLA-DPA1*02:01/DPB1*01:01 and the HTL epitope ERTRFQTLLNELDRS are listed as examples. Colored lines, each representing a unique interaction, connect interacting amino acids.

The HLA peptide-binding structure comprises a groove formed from two antiparallel α-helices overlying an eight-stranded β-sheet. The peptide-binding groove of class II MHC molecules is a heterodimer, with constituent chains such as HLA-DPA1*02:01 and HLA-DPB1*01:01 [129]. MHC-II molecules may exist as heterodimers (e.g., HLA-DPA1*02:01/DPB1*01:01) or homodimers (HLA-DRB1*04:05). Molecular docking is employed to forecast the optimal orientation of one molecule when it forms a stable complex with another. Molecular analysis of HLA and HTL epitope docking was conducted employing AlphaFold3. In all complexes, for the HLA region of two α-helices and the groove between them, pLDDT values were >90. Molecular docking interactions were visualized and quantified via the PBDSum web server. In all cases, binding of 15-mer peptides was observed in the HLA groove region (Figure 2).

The PDBsum service was used to obtain information about interacting amino acid residues at the protein–protein interface. Binding strength is determined by the length of the interface region and the number of residues present. A greater proportion of amino acids in longer interface regions indicates stronger binding and more stable conformations. The interaction between HTL epitopes and HLA molecules was found to involve 9–19 hydrogen bonds and 0–4 salt bridges (Figure 2, Table 3). These hydrogen bonds and salt bridges play a central role in stabilizing the interaction between the HTL epitope and HLA [130]. The greatest number of linkages was observed between the following pairs: HLA-DRB1*04:05 + AERTRFQTLLNELDR, HLA-DPA1*02:01/DPB1*05:01 + AERTRFQTLLNELDR, HLA-DPA1*01:03/DPB1*02:01 YSEILDKYFKNFDNG, and HLA-DRB1*04:01 + ERTRFQTLLNELDRS. The fewest observed linkages were those between HLA-DPA1*02:01/DPB1*14:01 + RERKVEAEVQAIQEQ, HLA-DRB1*15:01 + TIRIGIYIGAGICAG, and HLA-DRB1*07:01 + PQSAVYSTGSNGILL. Consequently, a significant correlation between the HTL epitope and HLA was observed in every instance.

We assessed the binding affinity of 15-mer peptides to HLA-II using Gibbs free energy (ΔG) and dissociation constant (M) calculations from molecular docking via the PRODIGY web server [131,132]. Table 4 presents a summary of the data. The range of peptide–HLA allele binding energies was −9.3 to −13.9 kcal/mol. All HLA-II molecules demonstrated efficient interaction with their cognate predicted HTL epitopes. The Gibbs energy values determined in this study were found to agree with those reported in prior investigations. For example, binding energies between seven experimentally confirmed antigenic epitopes of poxvirus and HLA-A*02:01 molecules ranged from −9.3 to −12.9 kcal/mol [133]. The PRODIGY server predictions of peptide–receptor binding affinity suggest strong binding for each peptide. The substantially negative Gibbs free energy change (ΔG) and the low dissociation constant (Kd) are indicative of a robust and stable interaction. The highest binding efficiency was identified for HLA-DRB1*04:01 + ERTRFQTLLNELDRS, HLA-DPA1*01:03/DPB1*02:01 + YSEILDKYFKNFDNG, and HLA-DRB1*04:05 + AERTRFQTLLNELDR.

### 2.4. Molecular Dynamics Simulations of the HTL–Epitope Complex and HLA-II

Molecular dynamics (MD) simulations were conducted to assess the stability of docked MHC II complexes and their corresponding predicted HTL epitopes. The stability of the HTL–epitope/HLA complex and the extent of its conformational dynamics can be evaluated by analyzing the RMSD changes observed during the simulation. The peptides demonstrated conformational stability within the binding pocket upon stable epitope binding, as evidenced by internal oscillations that remained within a 2 Å deviation throughout the simulation and lacked significant structural perturbation (Figure S1). Stable hydrophobic and polar interactions were observed in each complex for over 30% of the simulation time (Table 5). Notably, a subset of interactions demonstrated high stability, persisting throughout over 90% of the simulation. Specifically, ASP 237 (HLA-DRB1*04:01 B-chain) displayed two polar bonds with the ligand, present for 97% and 80% of the simulation, while ASN 68 (HLA-DRB1*04:01 A-chain) exhibited two polar bonds with persistence exceeding 99% and 96%, respectively.

## 3. Discussion

Viral infections are potential causative agents in the development of autoimmune diseases [134]. One process that can lead to autoreactivity is known as molecular mimicry [135,136]. Pathogens can “mimic” the protein structures of their host to evade the immune response [137]. Although the body possesses defense mechanisms to prevent autoimmunity, the phenomenon of molecular mimicry may induce cross-reactivity in T and B lymphocytes, targeting both the mimicking and the mimicked antigens. Such cross-reactivity may be fundamental to the pathogenesis of diverse autoimmune pathologies [2,138,139]. Similar to other viruses, SARS-CoV-2 may induce autoimmune processes post-infection [140,141]. For example, a correlation has been established between COVID-19 and the onset of autoimmune diseases, specifically Guillain–Barré syndrome [142], Miller–Fisher syndrome [143], immune thrombocytopenic purpura [144], and autoimmune hemolytic anemia [145].

The role of molecular mimicry in the etiology of autoimmune diseases has been confirmed by various findings. For example, several studies have demonstrated an increased risk of developing autoimmune pathologies after various infections [9,146,147]. Also reported in the literature were elevated autoantibody titers in patients after infections [148] and cross-reactivity among antibodies and lymphocytes in response to viral antigens and autoantigens [13,149,150]. Furthermore, the immunization of animals with viral proteins has been demonstrated to exacerbate the severity of human disease models [151,152].

SARS-CoV-2 infection may trigger cross-reactivity with host autoantigens, leading to the production of various autoantibodies, including antiphospholipid, antinuclear, and antibodies targeting annexin A2, ACE2, and other tissue and immune system components [21]. Antiphospholipid autoantibodies, associated with a risk of thrombophilia, were detected in COVID-19 patients, ranging from 24% to 57% [153,154,155]. Furthermore, the following autoantibodies were identified in patients with confirmed cases of SARS-CoV-2: anti-erythrocyte antigens [156], anti-neutrophil cytoplasmic antibodies [153], and antibodies to cyclic citrullinated peptide [157]. Neurological manifestations in COVID-19 patients have been associated with the presence of autoantibodies against contactin-associated protein 2 (anti-Caspr2), GD1b ganglioside (anti-GD1b), myelin oligodendrocyte glycoprotein (anti-MOG), and myelin basic protein (anti-ObM) [85,86,87]. It is thus highly probable that SARS-CoV-2 infection triggers the production of autoantibodies that recognize a multitude of intrinsic proteins.

The molecular mimicry hypothesis is supported by a small but growing body of recent research demonstrating sequence similarity between SARS-CoV-2 and human proteins [158,159,160]. It is important to note that most of the aforementioned studies omitted an analysis of antibody binding to human protein sequences [161,162,163]. This study investigated the presence of homologous sequences within human protein epitopes and their potential recognition by the human immune system.

The analysis of epitopes in different proteins relies on the concept of short compound sequences (SCSs) comprising amino acids from proteins, with the primary sequence of a protein fragmented into peptides that are offset from each other by one amino acid residue [164,165,166,167,168]. It is assumed that antibodies recognize epitopes with at least five amino acids [169,170]. However, a number of studies on protein epitope mapping have used longer sequences, such as seven or more amino acids [171,172,173,174,175]. The choice of peptide length is due to the need to balance sensitivity and specificity in their analysis. Longer peptides can be more specific to a particular antigen, while shorter peptides can be more sensitive but less specific. Therefore, in our study, we settled on sequences of seven amino acids.

The primary sequence of the SARS-CoV-2 S protein was divided into heptapeptides (SCS), and the resulting heptapeptide library was screened for sequence homology against the human proteome. The analysis of 28 SCSs revealed the presence of human proteins with identical amino acid sequences. However, identified sequence homologies do not necessarily indicate immune response, as structural constraints or subcellular localization may prevent these regions from functioning as actual epitopes. To identify SCS sequences potentially capable of eliciting an immune response, B-cell epitope prediction was conducted for each selected human protein exhibiting sequence homology. Our computational analysis identified surface-accessible regions in nine human proteins (DAAM2, CHL1, HAVR2/TIM3, FSTL1, FHOD3, MYO18A, EMILIN3, LAMP1, and αENaC) that exhibit sequence similarity to the SARS-CoV-2 S protein and are predicted to be potentially recognizable by B-cell receptors based on bioinformatic criteria. Our data are consistent with those obtained by high-throughput methods, which demonstrate a broad diversity of autoantibodies produced in COVID-19 patients [176,177,178].

Autoreactive B cells contribute to the initiation of inflammatory and immune responses through the production of excessive autoantibodies and pro-inflammatory cytokines, as well as their function as antigen-presenting cells, leading to subsequent tissue and organ damage in affected individuals [179]. It is noteworthy that autoantibodies can be detected years before the manifestation of clinical symptoms associated with autoimmune diseases [180,181]. Mechanisms of immune tolerance mitigate antibody-mediated autoimmunity in autoreactive B cells [182]. The mechanisms controlling B-cell development can be divided into central immune tolerance and peripheral immune tolerance [183]. A considerable fraction (20% to 40%) of autoreactive B cells circumvent central tolerance mechanisms, migrating to the periphery where they persist as transient, naïve B cells [184]. Peripheral immune tolerance mechanisms preferentially control the affinity maturation of autoreactive B cells, leading to the elimination of those exhibiting strong self-antigen reactivity by clonal inactivation and clearance. It has been shown that, on average, half of the B cells that bind both foreign and autoantigens are removed between the transitional and mature stages of B-cell maturation [185]. The surviving clones represent a B-cell repertoire with the capacity to produce autoreactive antibodies in response to viral infection [186]. Significantly, a complete absence of autoreactive, antibody-producing B cells is not achieved, with the level of elimination seemingly contingent upon the autoantigen’s properties [185]. A moderate degree of autoreactivity is considered crucial to B-cell development [187]. The precise mechanisms responsible for compromised immune tolerance require further elucidation. Autoimmune disease pathogenesis arises from an imbalance between B-cell immune tolerance and autoantibody production [188]. It cannot be excluded that activation of autoreactive B-lymphocytes capable of binding both to epitopes of the S protein of SARS-CoV-2 and to epitopes of their own proteins is involved in the development of autoimmune processes after COVID-19.

B-cell antigen presentation is crucial for activating CD4+ T cells, which orchestrate the immune response and further stimulate B-cell activity [115]. Nine human proteins bearing B-cell epitopes homologous to SARS-CoV-2 S protein heptapeptides were analyzed for potential helper T lymphocyte epitopes. For each protein, the predicted optimal HLA class II allele was identified using in silico methods. Bioinformatic docking analysis revealed a significant interaction between all peptides and HLA-II molecules. All HLA–epitope complexes exhibited robust binding, with the peptides ERTRFQTLLNELDRS, YSEILDKYFKNFDNG, and AERTRFQTLLNELDR demonstrating the strongest interactions.

In autoimmune diseases, specific HLA loci are often associated with an increased risk of developing these conditions [189]. For example, HLA-DRB1*04:01, 04:04, 04:05, and 01:01 alleles are associated with susceptibility to rheumatoid arthritis [190,191,192,193]. A correlation has been established between the HLA-DPB1∗05:01 allele and susceptibility to Graves’ disease [194]. Multiple sclerosis and myasthenia gravis have been associated with the HLA-DRB1*15:01 allele as a primary genetic risk factor [195,196]. Polymorphism in HLA alleles can either increase susceptibility to or confer protection against autoimmune disorders by modulating the peptide repertoire displayed and consequently influencing the adaptive immune response. The binding affinity of HLA alleles for peptides varies, leading to distinct surface peptide repertoires [197,198]. In this study, HLA class II alleles (HLA-DPA1*01:03/DPB1*02:01, HLA-DPA1*02:01/DPB1*01:01, HLA-DPA1*02:01/DPB1*05:01, HLA-DPA1*02:01/DPB1*05:01, HLA-DPA1*02:01/DPB1*14:01, HLA-DQA1*01:02/DQB1*06:02, HLA-DRB1*04:01, HLA-DRB1*04:05, HLA-DRB1*07:01, and HLA-DRB1*15:01) that can be associated with post-COVID-19 autoimmunity were predicted based on bioinformatics analysis. These findings underscore the significance of identifying disease-associated MHC/HLA-presented peptides for enhanced comprehension of autoimmunity.

Despite the limitations, this in silico study provides important details that expand our understanding of potential molecular mimicry mechanisms, shedding light on the possible role of autoreactive epitopes in the pathogenesis of virus-associated autoimmune reactions. The analysis of viral epitopes is crucial for vaccine development. The inclusion of sequences homologous to human proteins in vaccine epitopes is not recommended due to potential safety concerns. Therefore, the described bioinformatics analysis methodology can find practical application in vaccine development. The results obtained are also important for a better understanding of immunological and autoimmune processes as viral infection complications.

### Limitations of the Study

Our study is limited by the use of exclusively bioinformatic methods, which require further experimental verification. The data obtained are predictive in nature.

## 4. Materials and Methods

### 4.1. Obtaining the S Protein Sequence of SARS-CoV-2 and Predicting B-Cell Epitopes Using IEDB

The SARS-CoV-2 (Wuhan strain) S protein sequence (BCN86353.1) was downloaded from the National Center for Biotechnology Information (NCBI) (https://www.ncbi.nlm.nih.gov, accessed 2 December 2024).

SARS-CoV-2 Variant of Concern protein sequences were obtained from the ViralZone repository (https://viralzone.expasy.org/9556, accessed 2 December 2024): Alpha (B.1.1.7), Beta (B.1.351), Delta (B.1.617.2), and Omicron (B.1.1.529). Conserved regions within coronavirus S glycoproteins were identified via multiple sequence alignments utilizing Clustal Omega, accessible through the EMBL-EBI (https://www.ebi.ac.uk, accessed 2 December 2024).

The primary sequence of the S protein was divided into heptapeptides shifted by one residue (i.e., MFLLTTK, FLLTTKR, LLTTKRT, LTTKRTM, etc.) using Python 3.13. The resulting list of peptides was analyzed for homology with human proteins using the UniProtKB service (https://www.uniprot.org/peptide-search, accessed 3 December 2024) [199,200].

B-cell epitope prediction for the SARS-CoV-2 S protein was performed using the Immune Epitope Database resource (IEDB, www.iedb.org, accessed 4 December 2024) [201]. The selection process was limited to linear epitopes.

### 4.2. Obtaining Human Protein Sequences and Predicting B-Cell Epitopes Using ABCpred

We identified the regions of selected human proteins, homologous to the SARS-CoV-2 S protein, that can elicit an immune response. The protein sequence data were sourced from the UniProt database (https://www.uniprot.org/, accessed 17 December 2024). B-cell epitope prediction was performed using the ABCpred resource (https://webs.iiitd.edu.in/raghava/abcpred/, accessed 17 December 2024) [202,203]. Predicted B-cell epitopes were hierarchically ordered based on the scores generated by a trained recurrent neural network. A higher peptide score indicates a stronger probability for the peptide to be an epitope. At this stage, epitopes with a score > 0.7 were selected.

### 4.3. Predicting HTL (Helper T Lymphocyte) Epitopes

MHC-II binding predictions were generated using the IEDB tool (http://tools.iedb.org/mhcii/, accessed 11 January 2025) [204,205]. We used the Net MCpan prediction method [206]. A reference panel comprising seven alleles was selected for analysis, as detailed in [124]. HLA alleles were selected based on a rank of 2 or less. The predicted epitopes underwent a search to detect heptapeptide sequence occurrences.

HLA allele frequency data were acquired from the Allele Frequency Net Database (http://www.allelefrequencies.net/top10freqs.asp, accessed 23 January 2025).

### 4.4. Molecular Docking

Molecular docking analysis was conducted to assess the interaction between the HTL epitope and HLA molecules capable of recognizing it. HLA sequences were retrieved from the IPD-IMGT/HLA database (https://www.ebi.ac.uk/ipd/imgt/hla/, accessed 18 February 2025). Three-dimensional modeling of HLA structures was conducted using AlphaFold3 (https://alphafoldserver.com/, accessed 18 February 2025). The input data comprised chains A and B (HLA sequences) and chain C (epitope sequence). The modeling results were visualized using PyMOL 3.1.3.

PDBsum (https://www.ebi.ac.uk/thornton-srv/databases/pdbsum/, accessed 20 March 2025) [207] was utilized for binding analysis. Binding affinity, expressed as Gibbs free energy (ΔG) and dissociation constant (M) at 37 °C, was determined using the PRODIGY server (https://rascar.science.uu.nl/prodigy/ accessed 24 March 2025) [131,132].

### 4.5. Molecular Dynamics

Molecular dynamics simulations were conducted using methods detailed in previous studies [208,209]. The analysis process included the following steps: (1) importing the PDB file of the protein complex, (2) preparing the protein using the “protein preparation workflow” function, (3) separating chains and docking using the “Protein–Protein Docking” module, (4) selecting the optimal structure, (5) selecting solvation and ions parameters using the “System Builder” function, (6) molecular dynamics simulations, (7) trajectory analysis.

Proteins were prepared using the “protein preparation workflow” function with default settings. Hydrogen bonds were optimized and a final constrained minimization of the system was performed at pH 7.4, in the OPLS4 force field [210] with full optimization for hydrogen atoms and a maximum RMSD of heavy atoms from the initial position of 0.30 Å.

The MHC sequence (chains A and B) was separated from the peptide (chain C). Protein–protein docking was performed using the “Protein–Protein Docking” module. Chains A and B were selected as the receptor, and chain C as the ligand. The following settings were chosen: number of ligand rotations to probe—70,000; maximum poses to return—30. At the first stage, the generated poses were visually inspected and those where the peptide is located in the groove formed by the HLA chains were selected. Then, one structure was selected using the “Protein structure alignment” evaluation function.

The HLA complex, incorporating the relevant HTL epitope, was positioned within an orthorhombic crystal system, maintaining a 15 Å buffer from the protein surface. An aqueous solution of NaCl, at a concentration of 0.15 M, was introduced into the system. The TIP3P solvent model was utilized. The force field was OPLS4 [210]. The excess positive charges present on the protein were neutralized by the addition of sodium and chloride ions. Molecular dynamics simulations were executed over a 100-nanosecond period, with an integration step size of 2 picoseconds and a temperature of 310 Kelvin. The NPT ensemble method was implemented, incorporating a Martyna–Tobias–Klein barostat and a Nose–Hoover thermostat. A total of 1000 simulation frames were collected for statistical analysis. Computational analysis within this research utilized the SRF SKIF computing cluster prototype. The Desmond 7.2 program was utilized for all calculations.

## 5. Conclusions

In this study, we employed immunoinformatic approaches to analyze the potential molecular mimicry between the SARS-CoV-2 S protein and the human proteome. A comparative in silico analysis was conducted between the human proteome and S protein heptapeptides to identify regions of sequence homology. Surface-accessible regions were identified in nine human proteins that exhibit sequence similarity to the SARS-CoV-2 S protein and are predicted to be potentially recognizable by B-cell receptors. Our findings demonstrate that the proteins DAAM2, CHL1, HAVR2/TIM3, FSTL1, FHOD3, MYO18A, EMILIN3, LAMP1, and αENaC contain regions with sequence homology to the SARS-CoV-2 S protein. These homologous sequences represent potential candidates for cross-reactive immune recognition.

The activation of CD4+ T-cell responses is significantly influenced by B-cell-mediated antigen presentation. This study assessed potential CD4+ T-cell responses through computational prediction of HTL epitopes containing homologous sequences. Using molecular docking simulations, we assessed the binding capacity between identified HTL epitopes and HLA class II molecules. In all cases, a strong affinity between the peptide and HLA was observed due to hydrogen bonds and salt bridges. The range of peptide–HLA allele binding energies was −9.3 to −13.9 kcal/mol. Thus, the study identified ten epitopes and their associated HLAs (HLA-DPA1*01:03/DPB1*02:01 + YSEILDKYFKNFDNG, HLA-DPA1*02:01/DPB1*01:01 + ERTRFQTLLNELDRS, HLA-DPA1*02:01/DPB1*05:01 + AERTRFQTLLNELDR, HLA-DPA1*02:01/DPB1*14:01 + ERTRFQTLLNELDRS, HLA-DPA1*02:01/DPB1*14:01 + RERKVEAEVQAIQEQ, HLA-DQA1*01:02/DQB1*06:02 + NAINIGLTVLPPPRT, HLA-DRB1*04:01 + ERTRFQTLLNELDRS, HLA-DRB1*04:05 + AERTRFQTLLNELDR, HLA-DRB1*07:01 + PQSAVYSTGSNGILL, and HLA-DRB1*15:01 + TIRIGIYIGAGICAG) that represent potential candidates for cross-reactive T-cell responses based on our computational models. Furthermore, we identified seven autoepitopes (YSEILDKYFKNFDNG, ERTRFQTLLNELDRS, AERTRFQTLLNELDR, RERKVEAEVQAIQEQ, NAINIGLTVLPPPRT, PQSAVYSTGSNGILL, and TIRIGIYIGAGICAG) that may contribute to autoimmune pathogenesis when presented by class II HLA molecules. This in silico study provides important details that expand our understanding of potential mechanisms of molecular mimicry, shedding light on the possible role of autoreactive epitopes in the pathogenesis of virus-associated autoimmune reactions. The results obtained are also important for a better understanding of immunological and autoimmune processes.

## Figures and Tables

**Table 1:** Human proteins that have a site of homology with the heptapeptides of the SARS-CoV-2 S protein located in the B-cell epitope region.

Gene Names	ID UniprotKB	Heptapeptides	Autoepitope Containing the Region of Homology	Score ABCpred	Protein Localization
*ZNF528*	Q3MIS6	DKVFRSS	CHECDKVFRSSSKLAQ	0.71	Nucleus
*OTUD6A*	Q7L8S5	FLPFFSN	HVDEFLPFFSNPETSD	0.71	Cellular
*LAMP1*	P11279	VSGTNGT	SPSVDKYNVSGTNGTC	0.86	Transmembrane
*ABCA10*	Q8WWZ4	SLLIVNN	EQIPKTPLTSLLIVNN	0.91	Transmembrane
*DAAM2*	Q86T65	TRFQTLL	QVYAAERTRFQTLLNE	0.84	Extracellular
*BLTP1*	Q2LD37	SSSGWTA	SSSGWTAVGMENDKKE	0.90	Cellular
*CHL1*	NCHL1	YSTGSNV	QSAVYSTGSNGILLCE	0.77	Extracellular
*HAVCR2*	Q8TDQ0	IGAGICA	GIYIGAGICAGLALAL	0.84	Extracellular
*HPS1*	A0A0S2Z3U9	SPRRARS	DDIQPSPRRARSSQNI	0.89	Cellular
*SCNN1A*	C5HTY8	RRARSVA	HGARRARSVASSLRDN	0.84	Transmembrane
*TNK1*	Q13470	VTLADAG	SFPASAVTLADAGGLP	0.85	Cellular
*FHOD3*	A0A0A0MTS9	GLTVLPP	AINIGLTVLPPPRTIK	0.74	Extracellular
*EMILIN3*	Q9NT22	KVEAEVQ	ERKVEAEVQAIQEQVS	0.73	Extracellular
*MYO18A*	A0A994J771	LIRAAEI	ARLIRAAEINGEVDDD	0.90	Extracellular
*FSTL1*	Q12841	LDKYFKN	YSEILDKYFKNFDNGD	0.84	Extracellular
*THADA*	H0Y3V5	NASVVNI	HDSFDMKDLNASVVNI	0.72	Cellular
*SET*	A0A0C4DFV9	EIDRLNE	IEHIDEVQNEIDRLNE	0.80	Nucleus
*MYO16*	F8W883	EDDSEPV	GDEDDSEPVYIEMLGH	0.70	Cellular

**Table 2:** Incorporation of homologous heptapeptides into the HTL epitope sequences of 6 human proteins, and their corresponding HLA class II alleles.

HLA Class II	Start Position	SCS	HTL Epitope	Score	Rank
**DAAM2**					
HLA-DRB1*04:05	248	TRFQTLL	AERTRFQTLLNELDR	0.9254	0.2
HLA-DPA1*02:01/DPB1*05:01	248	TRFQTLL	AERTRFQTLLNELDR	0.1115	1.8
HLA-DPA1*02:01/DPB1*01:01	249	TRFQTLL	ERTRFQTLLNELDRS	0.1398	1.8
HLA-DRB1*04:01	249	TRFQTLL	ERTRFQTLLNELDRS	0.6160	1.9
HLA-DPA1*02:01/DPB1*14:01	249	TRFQTLL	ERTRFQTLLNELDRS	0.1356	2.0
**HAVCR2**					
HLA-DRB1*15:01	198	IGAGICA	TIRIGIYIGAGICAG	0.7440	0.7
**FHOD3**					
HLA-DQA1*01:02/DQB1*06:02	73	GLTVLPP	NAINIGLTVLPPPRT	0.4816	1.6
**CHL1**					
HLA-DRB1*07:01	337	YSTGSNV	PQSAVYSTGSNGILL	0.7595	0.7
**EMILIN3**					
HLA-DPA1*02:01/DPB1*14:01	622	KVEAEVQ	RERKVEAEVQAIQEQ	0.2145	0.7
**FSTL1**					
HLA-DPA1*01:03/DPB1*02:01	145	LDKYFKN	YSEILDKYFKNFDNG	0.6819	0.5

**Table 3:** Interface statistics data of binding between HTL epitopes and HLA class II obtained using the PBDSum web service.

HLA (Chain A, B) + Epitope (Chain C)	Chains	No. of InterfaceResidues	Interface Area (Å2)	No. of Salt Bridges	No. of Hydrogen Bonds	No. of Non-Bonded Contacts
HLA-DRB1*04:05 AERTRFQTLLNELDR (DAAM2)	A C	23:13	702:935	1	12	124
	B C	18:10	662:839	-	7	92
HLA-DPA1*02:01/DPB1*05:01 AERTRFQTLLNELDR (DAAM2)	A C	18:13	755:820	2	13	105
	B C	19:13	682:814	1	6	79
HLA-DPA1*01:03/DPB1*02:01 YSEILDKYFKNFDNG (FSTL1)	A C	16:13	729:790	-	9	92
	B C	19:11	615:762	4	9	114
HLA-DRB1*04:01 ERTRFQTLLNELDRS (DAAM2)	A C	22:13	754:903	2	11	104
	B C	16:12	731:784	1	7	86
HLA-DPA1*02:01/DPB1*01:01 ERTRFQTLLNELDRS (DAAM2)	A C	19:14	746:813	1	12	120
	B C	16:7	528:643	-	2	69
HLA-DQA1*01:02/DQB1*06:02 NAINIGLTVLPPPRT (FHOD3)	A C	18:13	665:759	-	7	83
	B C	17:12	601:718	-	7	69
HLA-DPA1*02:01/DPB1*14:01 RERKVEAEVQAIQEQ (EMILIN3)	A C	19:12	701:771	2	8	95
	B C	14:11	542:629	-	4	53
HLA-DRB1*07:01 PQSAVYSTGSNGILL (CHL1)	A C	15:10	614:654	-	4	57
	B C	17:13	566:718	-	8	81
HLA-DRB1*15:01 TIRIGIYIGAGICAG (HAVCR2)	A C	13:12	622:666	1	6	67
	B C	17:13	629:698	-	5	79
HLA-DPA1*02:01/DPB1*14:01 ERTRFQTLLNELDRS (DAAM2)	A C	17:12	608:727	1	4	88
	B C	17:9	604:731	1	5	69

**Table 4:** Gibbs free energy (ΔG) and dissociation constant (M) between docked molecules of 15-mer peptide and HLA II using PRODIGY web server.

Complex HLA-II and HTL Epitope	ΔG (kcal mol^−1^)	Kd (M) at 37 °C
HLA-DRB1*04:01 + ERTRFQTLLNELDRS (DAAM2)	−12.9	8.4 × 10^−10^
HLA-DPA1*01:03/DPB1*02:01 + YSEILDKYFKNFDNG (FSTL1)	−12.7	1.1 × 10^−9^
HLA-DRB1*04:05 + AERTRFQTLLNELDR (DAAM2)	−11.9	4.0 × 10^−9^
HLA-DQA1*01:02/DQB1*06:02 + NAINIGLTVLPPPRT (FHOD3)	−11.8	4.6 × 10^−9^
HLA-DPA1*02:01/DPB1*05:01 + AERTRFQTLLNELDR (DAAM2)	−11.8	4.7 × 10^−9^
HLA-DPA1*02:01/DPB1*14:01 + ERTRFQTLLNELDRS (DAAM2)	−11.5	7.8 × 10^−9^
HLA-DPA1*02:01/DPB1*14:01 + RERKVEAEVQAIQEQ (EMILIN3)	−11.2	1.3 × 10^−8^
HLA-DPA1*02:01/DPB1*01:01 + ERTRFQTLLNELDRS (DAAM2)	−10.9	1.9 × 10^−8^
HLA-DRB1*15:01 + TIRIGIYIGAGICAG (HAVCR2)	−10.8	2.6 × 10^−8^
HLA-DRB1*07:01 + PQSAVYSTGSNGILL (CHL1)	−9.3	2.6 × 10^−7^

**Table 5:** Summary of contacts recorded during molecular dynamics simulations. The depicted interactions represent those exceeding 30% of the simulated trajectory (0.00–100.00 ns).

Complex HLA-II and HTL Epitope	Charged	Hydrophobic	Polar	Other Interactions
HLA-DRB1*04:01 + ERTRFQTLLNELDRS	B: ASP 237 (97%) B: ASP 237 (80%) * B: GLU 189 (68%) A: ARG 75 (62%) A: GLU 54 (48%) B: ASP 208 (34%) A: ASP 65 (33%) A: ARG 75 (31%) B: LYS 251 (30%)	B: TRP 241 (75%) B: TYR 210 (71%) B: TYR 212 (33%)	A: ASN 68 (99%) A: ASN 68 (96%) A: ASN 61 (72%) A: ASN 61 (67%) B: HIS 193 (59%) A: ASN 61 (39%)	
HLA-DPA1*01:03/DPB1*02:01 + YSEILDKYFKNFDNG	A: ARG 107 (88%) B: GLU 55 (87%) B: GLU 98 (85%) B: ASP 84 (73%) B: GLU 98 (56%) B: ARG 104 (51%) B: GLU 98 (47%) B: GLU 55 (47%) A: GLU 86 (40%) A: GLU 86 (40%) B: ARG 104 (33%) A:ARG 107 (32%)	B: TRP 88 (90%) B: TRP 88 (63%)	A: ASN 100 (99%) A: ASN 100 (98%) B: HIS 108 (48%) A: ASN 99 (31%) A: ASN 99 (30%)	
HLA-DRB1*04:05 + AERTRFQTLLNELDR	A:GLU 38 (95%) B:ARG 100 (82%) A:GLU 38 (78%) A:ARG 100 (50%) B:ARG 100 (30%)	B: TRP 90 (64%) B: TYR 59 (47%) B: TYR 76 (45%) B: TRP 90 (40%) A: TYR 89 (36%) A: TYR 89 (33%)	B:ASN 111 (64%)	
HLA-DQA1*01:02/DQB1*06:02 + NAINIGLTVLPPPRT	A: ASN 57 (95%) B: GLU 255 (39%) A: ASN 57 (34%) B: ARG 269 (33%)	B: TRP 65 (95%) B: TYR 211 (62%) B: TYR 211 (44%) B: TYR 211 (39%)	A: ASN 64 (88%) A: ASN 71 (56%) A: ASN 64 (55%)	A: GLY 55 (93%) A: GLY 55 (54%)
HLA-DPA1*02:01/DPB1*05:01 + AERTRFQTLLNELDR	B: GLU 315 (98%) B: LYS 358 (82%) B: GLU 357 (77%) B: GLU 357 (70%) A: GLU 86 (65%) B: GLU 357 (65%) B: LYS 358 (47%) B: LYS 358 (43%) A: GLU 86 (41%)	B: TYR 317 (99%) B: TRP 348 (82%)	A: ASN 93 (99%) A: ASN 100 (89%) B: GLN 302 (83%) A: ASN 100 (82%) B: GLN 302 (61%)	B: TYR 317 (73%)—Pi-Pi stacking
HLA-DPA1*02:01/DPB1*14:01 + ERTRFQTLLNELDRS	B: GLU 357 (72%) B: LYS 358 (70%) B: ARG 364 (54%) B: ARG 364 (50%) B: GLU 357 (48%)	B: TYR 317 (62%) B: LEU 354 (59%)	B: GLN 351 (79%) B: GLN 302 (46%) B: GLN 351 (43%) A: ASN 93 (37%)	A: GLY 98 (31%)
HLA-DPA1*02:01/DPB1*14:01 + RERKVEAEVQAIQEQ	A: GLU 86 (53%) B: GLU 357 (34%)	A: TYR 40 (80%) A: ALA 82 (80%) A: PHE 83 (48%)	A: SER 84 (99%) A: SER 84 (99%) B: ASN 369 (98%) A: ASN 93 (45%) B: GLN 302 (42%)	A: GLU 80 (42%)
HLA-DPA1*02:01/DPB1*01:01 + ERTRFQTLLNELDRS		B: TYR 207 (88%) A: TYR 9 (84%) B: TYR 209 (79%) B: TYR 209 (79%) B: TYR 214 (38%) B: TRP 238 (37%) B: TRP 238 (34%) B: TYR 214 (32%)	A: SER 53 (97%) A: ASN 62 (96%) B: HIS 258 (88%) A: SER 53 (82%) A: ASN 69 (77%) A: ASN 69 (73%) A: ASN 62 (49%) B: GLN 192 (49%) A: SER 53 (40%) B: ASN 259 (36%)	
HLA-DRB1*15:01 + TIRIGIYIGAGICAG	A: ASP 430 (97%) B: ARG 573 (59%) B: ARG 557 (50%) B: LYS 683 (48%) B: ASP 572 (38%) B: ARG 557 (37%) A: GLU 419 (32%)	A: PHE 415 (50%) B: PRO 555 (39%) B: TYR 574 (38%) B: PRO 555 (33%)	B: ASN 626 (100%) A: SER 417 (96%) A: SER 417 (95%) A: ASN 426 (82%) A: ASN 426 (54%) B: ASN 626 (44%) A: GLN 373 (41%) B: GLN 614 (33%) A: ASN 433 (31%) A: GLN 373 (31%) A: GLN 373 (30%) B: ASN 626 (30%)	B: HIS 625 (35%)—Pi-Pi stacking
HLA-DRB1*07:01 + PQSAVYSTGSNGILL	A: GLU 330 (52%) A: ASP 99 (50%) A: ASP 99 (47%)	B: TYR 327 (91%) A: TYR 42 (78%) B: LEU 348 (47%) A: TYR 152 (44%) B: TRP 304 (30%)	B: ASN 328 (46%) B: THR 372 (46%) B: ASN 377 (45%) B: ASN 377 (40%)	

* Interactions exceeding 100% are possible due to multiple interactions of a single type between some residues and the same ligand atom.

## Data Availability

The raw data supporting the conclusions of this article will be made available by the authors upon request.

## Supplementary Materials

The following supporting information can be downloaded at https://www.mdpi.com/article/10.3390/ijms26188793/s1.
