# Supplementary material for: SARS-CoV-2 Spike Protein and Molecular Mimicry: An Immunoinformatic Screen for Cross-Reactive Autoantigen Candidates
Source: Int J Mol Sci. 2025 Sep 10;26(18):8793. doi: 10.3390/ijms26188793 (PMC12469275; doi:10.3390/ijms26188793)

Figure S1. Fluctuation of the root mean square deviation (RMSD) values of atoms in the complex HLA-II and HTL epitope.

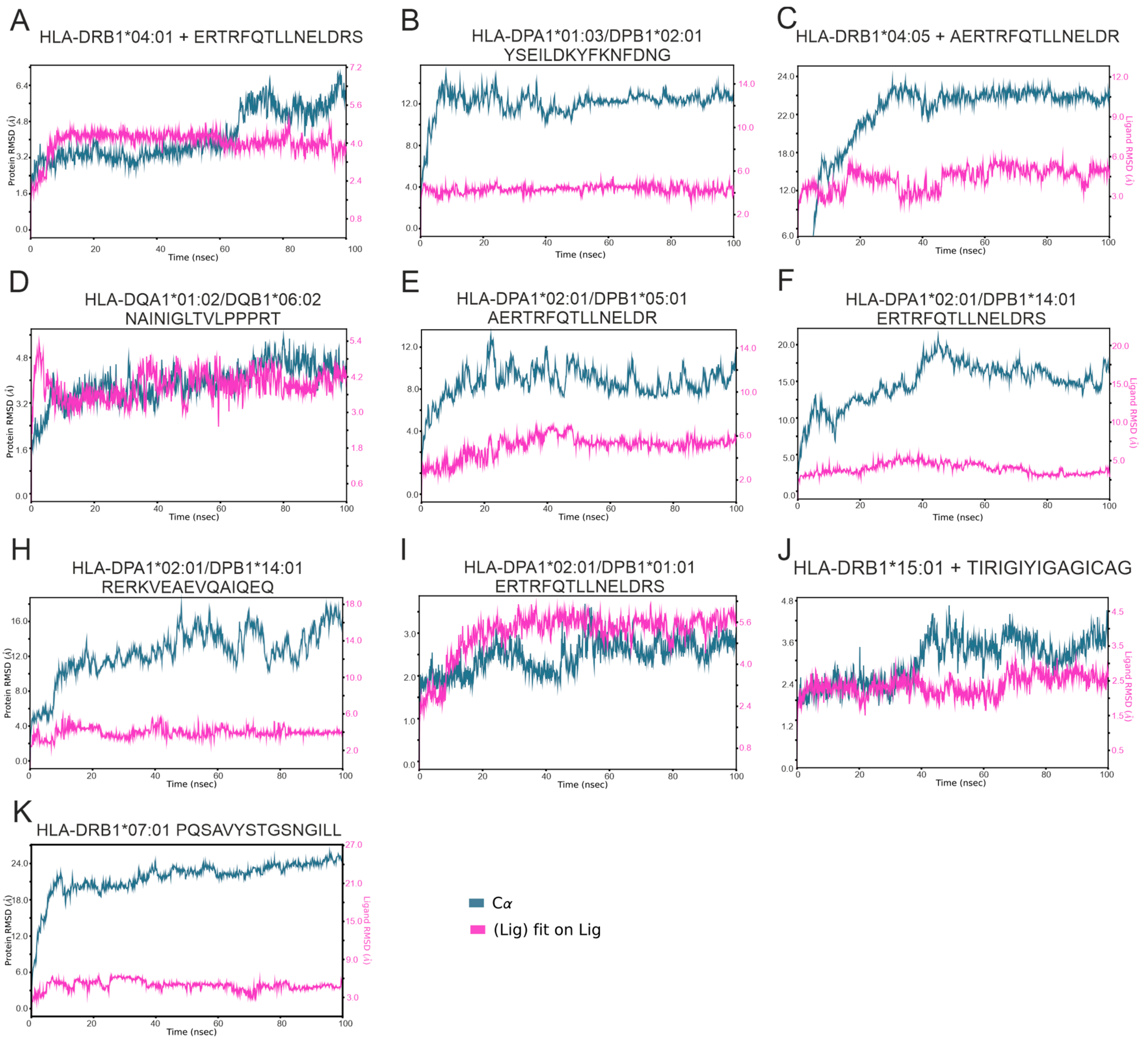

Supplement: Supplementary file 1 [file ijms-26-08793-s001.zip › ijms-3786238-supplementary/ijms-3786238-Figure S1.pdf]
